# An Acetazolamide Based Multimodal Analgesic Approach Versus Conventional Pain Management in Patients Undergoing Laparoscopic Living Donor Nephrectomy

**Published:** 2009-08

**Authors:** Rupinder Singh, Indu Sen, Jyotsna Wig, M Minz, Ashish Sharma, Indu Bala

**Affiliations:** 1 Senior Resident, Department of Anaesthesia & Intensive care, and Department of Transplant Surgery*, Post Graduate Institute of Medical Education & Research, Chandigarh, India, 160012; 2 Associate Professor, Department of Anaesthesia & Intensive care, and Department of Transplant Surgery*, Post Graduate Institute of Medical Education & Research, Chandigarh, India, 160012; 3 Professor & Head, Department of Anaesthesia & Intensive care, and Department of Transplant Surgery*, Post Graduate Institute of Medical Education & Research, Chandigarh, India, 160012; 4 Professor & Head*, Department of Anaesthesia & Intensive care, and Department of Transplant Surgery*, Post Graduate Institute of Medical Education & Research, Chandigarh, India, 160012; 5 Assistant Professor*, Department of Anaesthesia & Intensive care, and Department of Transplant Surgery*, Post Graduate Institute of Medical Education & Research, Chandigarh, India, 160012; 6 Professor, Department of Anaesthesia & Intensive care, and Department of Transplant Surgery*, Post Graduate Institute of Medical Education & Research, Chandigarh, India, 160012

**Keywords:** Laparoscopic surgery, Living donor nephrectomy, Multimodal analgesia, Acetazolamide

## Abstract

**Summary:**

Choice of an appropriate anaesthetic technique and adequate pain relief during laparoscopic living donor nephrectomy (LDN) is likely to make the procedure more appealing to kidney donors. Various analgesic regimens proposed to relieve pain after laparoscopic surgery include: opioids, non-opioid analgesics followed by opioids for the breakthrough pain and intra-peritoneal normal saline irrigation and instillation of local anaesthetics at surgical sites. Thorough literature review and medline search did not reveal any study where a combination of orogastric acetazolamide along with intraperitoneal saline irrigation and bupivacaine instillation techniques have been tried in these patients. In a prospective, double blind, randomized trial, eighty healthy adults undergoing LDN under general anaesthesia were enrolled to compare the efficacy of an acetazolamide based multimodal analgesic approach (Group A) with conventional pain management (Group B). Donors' demographics, intra-operative variables, early allograft function and recovery characteristics were evaluated for 72 hours. The primary end points were postoperative pain intensity on a visual analog scale and the incidence of shoulder tip pain (STP). The secondary end points included the latency of the rescue analgesia request rate, total analgesic consumption and patient satisfaction. Consistently lower mean pain scores were observed in Group A (*p*<0.03 for visceral pain). Frequency as well as the total dose of rescue analgesics administered was significantly less in Group A (*p*=0.001). Twelve patients (30.7%) in Group B complained of STP compared to three (7.5%) in Group A (*p*=0.025). Shoulder pain also presented earlier (8 hours versus 12 hours) and persisted for longer period in Group B (72 hours versus 48 hours, p 0.025).

To conclude, a multimodal analgesic approach consisting a combination of orogastric acetazolamide, intraperito-neal saline irrigation and use of bupivacaine in the operated renal fossa, pfannenstiel incision and laparoscopic port sites provide significant reduction in postoperative pain after LDN.

## Introduction

Laparoscopy has brought a substantial change in the field of renal transplantation with a gradual shift from the traditional laparotomy approach to a minimally in vasive laparoscopic nephrectomy technique^1^‐^4^ Although laparoscopic surgery facilitates a significantly faster recovery without compromising graft function, the CO_2_ pneumoperitoneum and patient positioning required for urology laparoscopy induces patho-physiological changes that makes the management of anaesthesia complex and challenging^3^‐^6^. Moreover, laparoscopic surgery still involves a painful recovery. Pain at the laparoscopic port sites, lower abdominal incision, pelvic organ nociception, ureteric colic and shoulder-tip pain contribute to the total pain experience in the postoperative period^7^‐^11^ Some patients may require more analgesia as compared to open nephrectomy in first 24 hours^2^.

Various analgesic regimens have been proposed to relieve pain after laparoscopy. These include: administration of oral opioids at regular intervals, non-opioid analgesics followed by opioids for the breakthrough pain and intravenous morphine infusion pumps for patient-controlled analgesia.^5^,^9^ Intra-peritoneal normal saline irrigation and instillation of local anaesthetics has been found to be effective in reducing the postoperative narcotic requirement.^12^ Alternatively, carbonic anhydrase inhibitors have been used to prevent the formation of carbonic acid.^13^,^14^ However, search for idealanalgesic regimens is still on. Since the aetiology of post operative pain following laparoscopic living donornephrectomy (LDN) is multi factorial and there is paucity of data on the multiple prong therapy in these patients. We planned this study to compare the analgesic efficacy of a combination of orogastric acetazolamide, intraperitoneal irrigation of normalsaline followed by instillation of bupivacaine in the operated renal fossa and bupivacaine infiltration at incision sites with the conventional care group, where only bupivacaine was infiltrated at incision sites in the patients undergoing laparoscopic donor nephrectomy. The primary end points of the study were postoperative pain intensity on a visual analog scale and the incidence of shoulder tip pain. The secondary end points included the latency of the rescue analgesia request rate, total analgesic consumption and patient satisfaction.

## Methods

After obtaining approval from the institutional ethics committee and written informed consent from the participants, this prospective, double blind, randomized trial was conducted on eighty healthy renal donors of either gender, ASAI-II, aged 18-55 years undergoing laparoscopic donor nephrectomy under general anaesthesia from July 2005-September 2007. During preanaesthetic evaluation, the participants were made familiar with 11 point visual analog scale. (where 0 is no pain and 10 is worst imaginable pain)^15^. We excluded patients with pre-existing neuromuscular disorders, shoulder pathology, chronic obstructive pulmonary disease, double renal artery, hypokalemia /hyponatremia/metabolic acidosis, sulfonamide allergy, diuretics or lithium therapy, analgesics/antiemetics intake in the last l2 hours. Donors undergoing removal of right kidney or patients in whom laparoscopic procedure had to be converted to open nephrectomy were not evaluated. All the participants were instructed to fast for eight hours prior to surgery. Premedication consisted of oral ranitidine (150mg), metoclopramide (10mg) & diazepam (5mg) administered two hours prior to surgery.

All participants were randomly allocated into two groups A & B, (n=40 each group) to receive either of the two analgesic regimens. Group A (multimodal analgesia care group) received orogastric acetazolamide through Ryle's tube soon after the induction of anaesthesia(5mg.kg^−1^ diluted in l0ml normal saline followed by 10 ml saline flushing). Powdered sachets of 5, 10, 50 and 100 mg acetazolamide were prepared with the help of microbalance for adequate dosing. At the completion of surgical procedure, 15-20 ml.kg^−1^ normal saline was used for the intrap eritoneal irrigation. This was followed by local instillation of 1.5mg.kg^−1^ dose of 0.5% bupivacaine in the operated renal fossa and bupivacaine infiltration (15ml of 0.25%) at the organ retrieval incision site and laparoscopic port sites. In Group B (Conventional care group) only bupivacaine (15ml of 0.25%) was infiltrated at surgical incision sites on the completion of procedure.

On the day of surgery, heart rate (HR), electrocardiography (ECG), arterial oxygen saturation (SpO_2_), non-invasive blood pressure (NIBP) and endtidal carbon dioxide (EtCO_2_) were monitored continuously and recorded at an interval of 10 minutes till the end of surgery. General anaesthesia was induced with intravenous morphine sulphate (0.15mg.kg^−1^), sleep dose of propofol (2 to 2.5mg.kg^−1^) and vecuronium (0.1mg.kg^−1^) to facilitate endotracheal intubation. Anaesthesia was maintained with Datex Ohmeda Aesitva-5 anesthesia ventilator using 100% oxygen and isoflurane (0. 5-2%) titrated to effect. After the induction of anaesthesia, a Ryle's tube was inserted orally and gastric contents were aspirated out. Acetazolamide 5mg.kg^−1^ diluted in 10ml normal saline was administered through Ryle's tube in Group A. Thereafter, donor was shifted to the modified flank position with the torso in a 45-degree lateral decubitus position for transperitoneal nephrectomy. Pneumoperitoneum was established by CO_2_ insufflation limiting pressure to <15mmHg. The total flow of carbon dioxide insufflated for producing pneum opertioneum was recorded. Intravenous ondansetron (100μg.kg^−1^) and intravenous morphine 3mg was administered half an hour before the expected completion of surgery in both groups. On the completion of procedure, neuromuscular blockade was reversed with intravenous neostigmine 50μg.kg^‐1^ and atropine 50μg.kg^‐1^. Patients' were extubated on meeting the standard criteria for extubation and shifted to renal post anaesthesia care unit (PACU). An anaesthesiologist who was not aware of the patients' group assignments recorded vital signs (heart rate, respiratory rate and non-invasive blood pressure), level of sedation, (assessed by the Modified Observers Assessment of Alertness/Sedation Score (OAA/S)^16^ and intensity of pain (assessed by a linear Visual Analog Scale)^15^ for the first 72 hours after completion of surgery. He recorded parietal and visceral pain at rest (supine), on movement (sitting up from supine) and after coughing. Shoulder pain was also evaluated. Pain assessments were done at 30 min, 2, 4, 8, 12, 24, 48 and 72 hours after shifting the patient to post anaesthesia care unit. Patients were requested to evaluate their overall postoperative pain management at the end of study period.

Rescue analgesia (intravenous injection of tramadol 1.5mg.kg^−1^) was given if VAS score was >3. If the pain persisted even after 30 minutes of intravenous tramadol administration, the single dose of intravenous pethidine 0.5-lmg.kg^−1^ was given (second rescue analgesic agent). The time from extubation of the patient to the administration of first dose ofrescue analgesic was recorded. Total dose and frequency of administration of tramadol and pethidine during the postoperative period were noted. The incidence and severity of postoperative nausea & vomiting/retching and the frequency of administration of rescue antiemetics were also noted. Side effects attributable to the study drug were specifically observed & recorded. (allergic reactions, drowsiness, paresthesia).

The number of patients required for the study were calculated to detect a difference of at least two pain scale units in aten point VAS. A total of 37 participants were needed to detect a significant difference between groups with a 0.05 level and 80% power in two-sided test of hypothesis. Adjusting for participants who may not complete the study, we enrolled 40 adults in each group. The demographic data and haemodynamic parameters were compared using independent t-test. Chi-square test was used to compare the descriptive data. Pain scores for the different pain components were compared using Mann Whitney ‘U’ test. The occurrence of postoperative emetic episodes, rescue antiemetic therapy and rescue analgesic therapy were analyzed with the Chi-square test or the Fisher Exact test where appropriate. The statistical analysis was performed using the SPSS for windows version 13.0. Statistical significance was defined as *p* = 0.05. All values were expressed as mean ± SD, median (IQR) or number (%).

## Results

Amongst eighty adults enrolled, one patient in the conventional care group required surgical re-exploration for post operative bleeding, hence he was excluded from data analysis. Donor characteristics, perioperative haemodynamic variables, mean EtCO_2_, duration of pneumoperitoneum, duration of surgery, anaesthesia time, quantity of intravenous fluids administered intra-operatively were comparable in boththe groups (Table 1).

**Table 1:** Donor Characteristics and perioperative data. *p*-value ≤ 0.05 is significant

Parameters	Group A(Mean±SD)	Group B(Mean±SD)	*p* Value
Age (Yrs)	41.93±10.49	42.73±9.86	0.72
Gender (M/F)	12/28	12/27	-
Weight (Kg)	58.93±0.01	60.23±0.91	0.57
Baseline Heart Rate (bpm)	84.58±0.8.54	83.18±10.52	0.51
Base Line SBP (mmHg)	124.45±12.07	127.25±10.11	0.26
Base Line DBP (mmHg)	78.25±9.06	76.80±8.33	0.49
Carbon Dioxide Flow (L)	195.30±35.132	194.33±31.762	0.89
Intra-Abdominal Pressure (mmHg)	12.30±0.5	11.30±1.0	0.70
Duration of Pneumoperitoneum (Min)	192.88±40.73	179.85±38.52	0.14
Duration of Surgery (Min)	235.60±38.57	235.08±36.51	0.90
Duration of Anaesthesia (Min)	252.73±38.444	255±37.50	0.70

SD-standard Deviation, SBP-Systolic blood pressure, DBP-Diastolic blood pressure, Date are mean± SD or n

**Fig 1:**
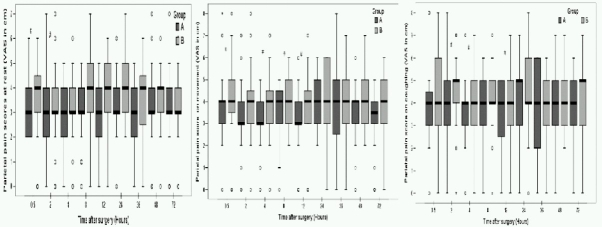
Box plots of postoperative parietal pain scores at rest, during movement and on coughing. Results are expressed in medians. The top and bottom of each box indicate 75^th^ and 25^th^ percentiles and the error bars 10^th^ and 90^th^ percentiles. O = outliers. # = p < 0.05.

**Fig 2:**
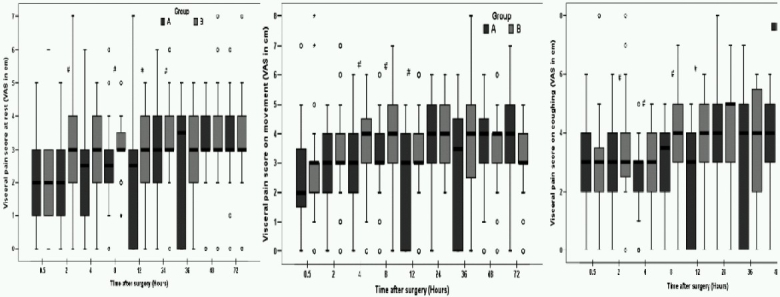
Box plots of postoperative visceral pain scores at rest, during movement and on coughing. Results are expressed in medians. The top and bottom of each box indicate 75^th^ and 25^th^ percentiles and the error bars 10^th^ and 90^th^ percentiles. O = outliers. # = p < 0.05.

Comparison of postoperative parietal and visceral pain VAS scores at rest, during movement and on coughing are depicted in Fig 1 & 2. Pain evaluations done at specific time intervals of 0.5, 2, 4, 8, 12, 24, 48 and 72 hours after extubation revealed that parietal pain was dominant over the visceral and shoulder pain in both the groups. However the intensity of pain was lesser on movement and coughing in multimodal analgesia group, especially during the first 12 postoperative hours. On adjusting forrepeated analysis of same variable over time, using the conservative bonferroni correction where p value of less than 0.006 was considered statistically significant, we found that at 48hr of interval the visceral pain at rest was less in Group A as compared to the Group B.

**Fig 3:**
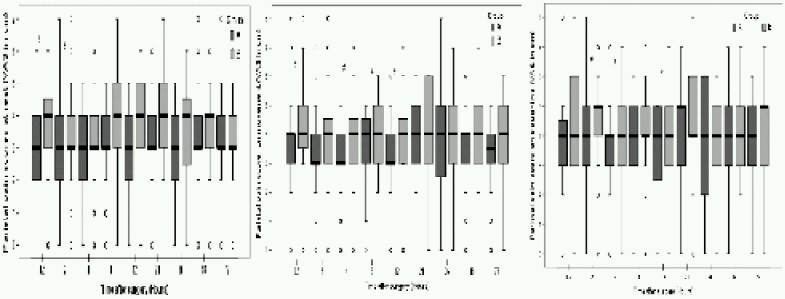
The mean (VAS in cm) intensity of parietal, visceral and shoulder tip pain (P 0.03) for visceral pain in group B.

Twelve patients (30.7%) in Group B complained of shoulder tip pain (STP) compared to 3 patients (7.5%) in Group A. (p=0.025). Pain also presented earlier in conventional care group (8hours) than in the multimodal analgesia group (12 hours). Assessment of pain at 36 postoperative hours indicated that 8(20.5%) patients in Group B had shoulder pain, whereas in Group A, none of participants complained of STP (p = 0.025). The pain also persisted up to 72hours in Group B (five patients; 12.8%) as compared to group A where only two patients complained of referred pain at 48 hours (Table 2). The mean intensity of shoulder tip pain (VAS) was lower in Group A compared to Group B at all time intervals in the postoperative period. This difference was statistically significant at 36 hrs and 72 hrs in the postoperative period (p = 0.05) (Table 3). The mean (VAS in cm) intensity of individual pain component ie parietal, visceraland shoulder tip pain are shown in Fig 3.

**Table 2:** Incidence and intensity of Shoulder tip pain after laparoscopic donor nephrectomy

Post extubation Time (hours)	Shoulder Tip Pain Number of patients (%)			Shoulder Tip Pain Scores Mean VAS (S.D)		
	Group A	Group B	*p*-value	Group A	Group B	*p*-value
0.5	0	0	–	0(0)	0(0)	1.00
2	0	0	–	0(0)	0(0)	1.00
4	0	0	–	0(0)	0.05(0.31)	0.31
8	0	1(2.5%)	1.00	0.33(0.99)	0.35(0.94)	0.77
12	1(2.5%)	0	1.00	0.45(1.01)	0.68(1.14)	0.39
24	1(2.5%)	3(7.6%)	0.35	1.15(1.44)	1.67(1.67)	0.29
36	0	8(20.5%)	0.002*	0.63(0.97)	1.77(1.77)	0.05*
48	2(5%)	6(15.3%)	0.15	1.23(1.38)	1.69(1.69)	0.06
72	0	5(12.8%)	0.025*	1.00(1.28)	1.67(1.67)	0.05*

p-value ≤0.05 is significant.

**Table 3:** Rescue Analgesia post LDN surgery (Mean ± SD)

Variables	Group A	Group B	P-value
Time of 1^st^ dose of first rescue analgesia (min)	189.30±152.28	122.30±88.46	0.045*
Total dose of tramadol (mg)	433.43±103.54	524.75±130.72	0.001*
Average no. of doses of tramadol	5.00±0.94	6.00±1.1	0.00*
Time of administration of second analgesia (min)	677.00±185.93	613.64±189.83	0.54
Total dose of 2^nd^ rescue analgesia (pethidine) (mg)	29±4.1	29±4.3	0.90
Second rescue analgesia: No. of patients (%)	5(12.5%)	11(27.5%)	0.08

* p-value<0.05 is significant

The time from extubation to the administration of first dose of tramadol was significantly longer in Group A (189.30± 152.28 min versus 122.30± 88.46 min Group B) (p=0.045). Both frequency and total consumption of tramadol were significantly less in Group A (p=0.00). The number of patients requiring second rescue analgesia, the difference in the frequency of administration and total dose of second rescue analgesia requirement was similar in both the groups. The second rescue analgesia (intravenous pethidine) was given in 5 patients (12.5%) of Group A versus 11 patients (27.5%) in Group B (p=0.08). From extubation, the time of administration of second analgesia was 677.00± 185.93 minutes in Group A and 613.64± 189.83 minutes in Group B (p=0.54). The total dose of pethidine administered postoperatively was similar in both the groups. (*p*=0.90) (Table 3).

The incidence of nausea was 27.5% in Group A and 51.2% in Group B (*p*=0.05). In Group A,7 (17.5%) patients had vomiting, while in Group B, 8 (20.5%) patients complained of vomiting in the postoperative period (*p*=0.95). Rescue antiemetics were given to 7 patients in Group A and 13 patients in Group B.No adverse effects were noted in any of the participants related to anaesthetic interventions. All the participants were satisfied with the anesthetic technique used.

## Discussion

Living donor nephrectomies are routinely being performed for last five years in our institute, thus fulfilling one of the basic criteria for design of perioperative analgesia trials. In the present study, two groups had similar demographic profile, perioperative hemodynamic parameters and other intraoperative variables like the duration of pneumoperitoneum, end tidal carbon dioxide concentration, surgery and anaesthesia time. As reported in the literature^5^,^7^‐^11^, the intensity of parietal pain perceived was more than visceral pain and pain used to aggravate during movement and coughing. However, we found consistently lower parietal and visceral pain scores and the incidence of shoulder pain was reduced to one fourth in Group A compared to Group B. This difference in pain scores can be attributed to the analgesic regimen used.

Previously, reduction in parietal pain scores have been demonstrated by local anaesthetics infiltration into the laparoscopic incision sites in laparoscopic chole-cystectomy, appendicectomy, gynecologic or urological laparoscopy patients.^5^,^7^,^17^‐^19^ However, the literature is not uniform on this aspect with several studies failing to show a significant effect.^20^,^21^ In a systematic review, Moiniche et al^20^ found no evidence of any measurable effect of port site infiltration with local anaesthetics on postoperative pain. In the present study, at the completion of procedure, all the patients received bupivacaine infiltration (15m1 of 0.25%) at surgical incision/port sites. But, Group B patients perceived significantly more pain, even during rest. There was a significant difference in median VAS on movement and coughingat 30 minutes, 4hour, 8hour and 12 hour of postoperative period. Thus, trocar site infiltration alone was not found to be effective for postoperative pain management. Another analgesic modality used in the treatment group was intraperitoneal saline irrigation for removal of residual carbon dioxide and bupivacaine instillation into the operated renal fossa. It has been reported that this maneuver significantly reduces postoperative analgesic requirements.^13^,^18^,^22^‐^27^ Recently, Boddy et al 12 conducted a meta-analysis of the 24randomized controlled trials to establish the safety and efficacy of intraperitoneal local anaesthesia in laparoscopic cholecystectomies. The drug was administered after the surgical dissection in fifteen trials and in another six studies, local anaesthetics were instilled both before and after the establishment of pneumoperitoneum. Authors suggested that local anaesthetics may be more effective if at least some of it is instilled before any surgical dissection. In present study, significant improvement in pain scores was noticed in the first 12 hours only. Further reduction in post-laparoscopic pain might have been achieved by preemptive administration of local anaesthetics. Future studies can be conducted to establish this fact in laparoscpic donor nephrectomies.

Patients in multimodalanalgesia group also received orogastric acetazolamide^13^, a carbonic anhydrase inhibitor which decreases the rate of formation of H^+^ ion and can retard peritoneal acidification responsible for visceral and referred pain after laparoscopy. Harvey et al^14^ investigated the effect of intravenous acetazolamide (5mg.kg^−1^) on post laparoscopic cholecystectomy pain and found that intravenous acetazolamide given just after induction of anesthesia reduces the referred pain in the initial postoperative period. In a previous study conducted in our institute (Bala I etal. Personal Communication), oral acetazolamide was administered two hours prior to laparoscopic cholecystectomy, incidence of STP was 35% in the control group, 15% in the acetazolamide group and 10% in the saline irrigation group. As IV preparation of the drug was not available in India and the bioavailability of drug is 100% even after oral use^13^, acetazolamide was administered via the orogastric route, just after the induction of anaesthesia. Using this technique concomitant with intraperitoneal saline irrigation and bupivacaine instillation reduced referred pain in multimodal analgesia group patients to 7.5% (72 hours observation period) though the duration of pneumoperitoneum was more than two times in LDN patients compared to laparoscopic cholecystectomy surgery. The reported incidence of shouldertip pain is 35-63% after laparoscopic sterilization^17^ and 30-45% post laparoscopic cholecystectomy^14^‐^21^,^22^, when patients were evaluated for 24-48 hours. Bisgaard et al observed an incidence of 38-66% in first week and 21-25 % in 4^th^ week after laparoscopic Nissen fundoplication.^28^ However, there is paucity of data on the incidence of shouldertip pain after laparoscopic renal surgeries. Keeping in mind, the nature of surgery and associated reduction in renal blood flow by pneumoperitoneum (which can predispose healthy renal donors to the postoperative risk of acute renal failure), intravenous tramadol was used to meet additional analgesia requirements and administration NSAID's drugs was avoided. The time interval for the first dose of rescue analgesiaadministration was longer and total analgesic consumption was reduced in multimodalanalgesia group.

Though present study is not adequately powered to detect drug-related side effects, none of the participants had adverse effects related to the study drugs (bupivacaine and acetazolamide). Sundaram et a1^29^ performed a retrospective chart review for 253 laparoscopic live donors. The overall rate of complications in the investigated series was 10.3%. Three of their patients required reex ploration for postoperative bleeding. In the present study, re-exploration was required in one of the participants where it was found that a weck clip had partially slipped from a gonadal vessel. This patient was excluded from the data analysis because repeat surgery potentially confounds postoperative pain. No other surgical complications were noted. All the allografts functioned well immediately after the surgery. There were no readmissions.

In conclusion, a multimodal analgesic approach provides betterpostoperative pain relief after LDN. This includes a combination of orogastric acetazolamide, intraperitoneal saline irrigation and use of bupivacaine in the operated renal fossa, pfannenstiel incision and laparoscopic port sites. Further large randomized trials are indicated to determine the cost-effectiveness and adverse event profile of this combined analgesia modality in laparoscopic donornephrectomy surgeries.
